# 1,4-Dihydropyridine Antihypertensive Drugs: Recent Advances in Photostabilization Strategies

**DOI:** 10.3390/pharmaceutics11020085

**Published:** 2019-02-17

**Authors:** Michele De Luca, Giuseppina Ioele, Gaetano Ragno

**Affiliations:** Department of Pharmacy, Health and Nutritional Sciences, University of Calabria, 87036 Rende, Italy; michele.deluca@unical.it (M.D.L.); giuseppina.ioele@unical.it (G.I.)

**Keywords:** 1,4-dihydropyridines, photostabilization, liposomes, cyclodextrins, light-absorbing excipients, opaque containers, nanosystems

## Abstract

The 1,4-dihydropyridine (DHP) drugs are nowadays the most used drugs in the treatment of hypertension. However, all the structures in this series present a significant sensitivity to light, leading to the complete loss of pharmacological activity. This degradation is particularly evident in aqueous solution, so much so that almost all DHP drugs on the market are formulated in solid preparations, especially tablets. The first and main process of photodegradation consists in the aromatization of the dihydropyridine ring, after which secondary processes can take place on the various substituents. A potential danger can result from the formation of single oxygen and superoxide species that can in turn trigger phototoxic reactions. Several strategies for the photostabilisation of DHP drugs have been proposed in recent years, in particular with the aim to formulate these drugs in liquid preparations, as well as to limit any toxicity problems related to light degradation. This review summarizes and describes the main aspects of the studies conducted in recent years to obtain photostable formulations of DHP drugs.

## 1. Introduction

The first of the 1,4-dihydropyridine (DHP) drugs, nifedipine, was synthesized in 1969 and approved by the Food and Drug Administration (FDA) in 1981. Given the great interest aroused by nifedipine, numerous congeners have been subsequently synthesized, with the main purpose of expanding the therapeutic effects and, at the same time, limiting the side effects and prolonging the half-life. During the 1990s, the use of these drugs surpassed that of verapamil, the first representative of the class of calcium antagonist antihypertensives, which had been introduced in therapy in the 1960s. Subsequently, DHP drugs have established themselves as the most prescribed antihypertensives [1]. The drugs for this pathology are currently classified as DHP and non-DHP derivatives, including among these the verapamil and diltiazem congeners [2], mainly used now as antiarrhythmics [3,4,5].

DHP drugs are inhibitors of L-type calcium-dependent calcium channels that are mainly spread outside the central nervous system. Particularly sensitive to DHP drugs are the L-channels present on the smooth muscle cells of the vessels, unlike those present in the heart, which are instead sensitive to diltiazem and verapamil. The hemodynamic effects of DHP drugs drugs are attributable to the dilatation at the arterial level which results in a reduction of peripheral resistance and of arterial pressure. Currently, the most used DHP drugs are those of the third generation, including amlodipine, barnidipine, lacidipine, lercanidipine, and manidipine. They are characterized by a long receptor half-life, due to the ability to rapidly abandon the blood flow to pass into the vessel wall, where they interact with the calcium channels. At the same time, they present reduced side effects.

**Figure 1:**
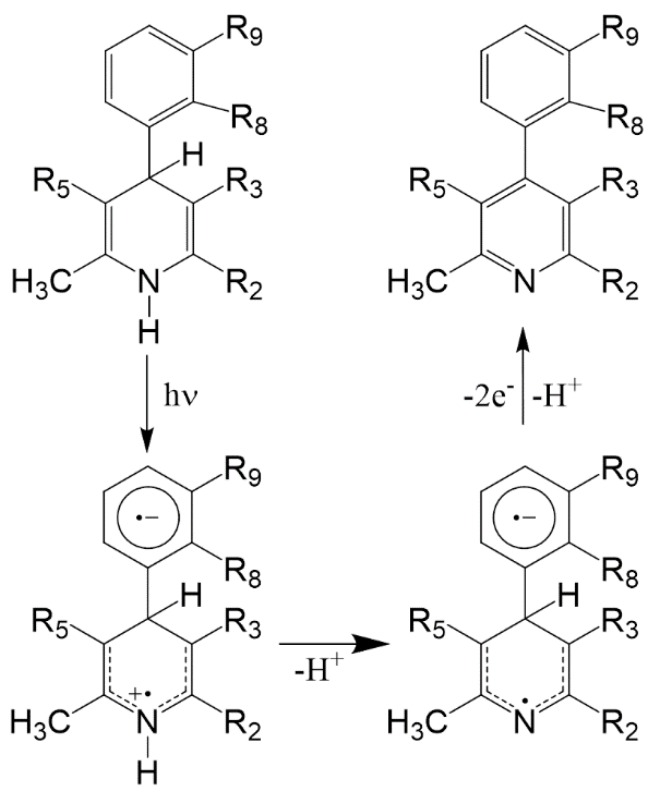
Hypothesized mechanism of the photodegradation process of 1,4-dihydropyridine (DHP) drugs causing potential phototoxicity.

All antihypertensive drugs with dihydropyridine structure have shown strict photolability. This vulnerability is to be held in high regard, as it involves loss of therapeutic effect and is potentially dangerous due to the probability of phototoxic reactions [6,7]. The chemical process of photodegradation generally consists in the oxidation of the dihydropyridine ring with the formation of the pyridine derivative. In some cases, the process leads to the formation of secondary photoproducts, mainly dependent on the functional groups present both on the phenyl and on the pyridine rings [8,9,10]. The mechanism starts with the transfer of the proton, probably to the solvent, from the excited single and it is favored, as in the case of nifedipine, by the presence of a nitro-group on the phenyl ring [11]. The photodegradation mechanism proposed is shown in Figure 1.

Analytical procedures to monitor the photodegradation of DHP drugs have been proposed in numerous papers and summarized in various reviews. Most of them use UV spectrophotometry and multivariate data analysis procedures [12,13,14,15,16,17,18]. Very interesting is a study where the photosensitivity of a series of DHP drugs is correlated to some molecular descriptors through a quantitative structure–property relationships (QSPR) model. This model, when applied to other DHP drugs, is able to predict their photodegradation rate [19].

One of the main problems related to the reaction of drugs to light is the possibility of causing skin phototoxic reactions, even after systemic administration. When the drug absorbs energy from light, it switches to an "energized" state with a high energy content. Returning to the ground state, free radicals or photoproducts, potentially toxic to cell membranes or DNA, can be formed [20,21]. For some DHP drigs, data and results on phototoxicity have been reported [6,7,10]. 

Nowadays, the development of a new drug must include the photostability study on both the product and the pharmaceutical formulations containing it. This aspect has been increasingly imposed over the years to ensure the quality and safety of commercial products, during manufacture and storage as well as during use by patients [22,23,24]. Since 1997, the International Council for Harmonisation (ICH) Technical Requirements for Pharmaceuticals for Human Use have detailed overall stress testing for the new drugs [25].

Alongside the studies on the chemical processes of photodegradation, on the kinetics of the photochemical reactions and, on the functional groups involved, a great interest has been aroused in studies that define new formulations or design technological systems capable of providing valid protection to the photosensitive drugs. In the case of DHP drugs, the photostabilization approaches have so far produced poor results, as DHP drugs are dispensed in solid formulations, usually tablets, where the stability is significantly high [26,27]. To our knowledge, currently only nifedipine and nimodipine are formulated in liquid preparations, packaged in dark glass containers or covered with opaque plastic material.

This review collects the results published in the last twenty years on the various approaches investigated for the photoprotection of DHP drugs. The paper is intended as a starting point for all operators in the field of pharmaceutical quality assurance. As sources of information, we have used standard research strategies that included the interrogation of the most famous databases of scientific articles using keywords, such as *dihydropyridines, photodegradation, photostabilization*, etc. as well as the evaluation of references of articles relevant to the topic. Some previously published papers refer to pioneering studies on the topic or are still today the only studies dedicated to the photostabilization of some DHP drugs.

## 2. Photostabilization Approaches

The pharmaceutical formulations of light-sensitive drugs can degrade both during production and storage or during use by patients. The knowledge of the photochemical behavior of these drugs is therefore necessary so that all precautions are taken for the handling of the active ingredients, the packaging phase and the drafting of the information of the package leaflet of the medicine. The European Pharmacopoeia indicates over 250 light-sensitive drugs and prescribes adequate protection, especially through the use of opaque or dark containers and appropriate packaging material [28].

The requirements for a photoprotective packaging strongly depend on the type of pharmaceutical preparation. This packaging very important for liquid formulations, where photodegradation reactions are favored, and has lesser significance for solid formulations. Indeed, 1,4-DHP series drugs are all formulated in solid preparations and only very few specialties are in a solution form: nimodipine in ethanol-polyoxyethylene hydrogenated castor oil, and nifedipine in polyethylene glycol. Both these solutions are packaged in amber glass bottles and the nifedipine bottle is wrapped in a black plastic coating.

In the last twenty years, when the degradation to light of drugs has become a research topic, the definition of photostabilization methods has gradually grown. Several approaches have been proposed and pharmaceutical companies now adopt some of them. Regarding the protection of DHP drugs, in the following paragraphs, the techniques that have received the most attention from the researchers and a good success in the application phase will be presented.

### 2.1. Use of Dark or Opaque Containers

The adoption of dark glass, or plastic containers, is still today the most commonly used method by the pharmaceutical industry to protect photosensitive drugs. Amber glass largely absorbs radiation in the ultraviolet region but has low filtering properties for infrared radiations. Green glass also has good protection against UV radiation. 

Most patients who take DHP drugs to treat hypertension are of old age and many of them are unable to swallow tablets or capsules, preferring the use of liquid formulations. The preparation of a liquid dosage form is also important for paediatric patients or for those adults who must receive medications via nasogastric or gastrostomy tube. Of course, one of the limits in the use of colored containers is their opacity or low transparency, which makes difficult to view the content. For this reason, research in recent years is turning to the study of alternative approaches. 

Hydroalcoholic solutions containing well-known DHP drugs (felodipine, lercanidipine, nimodipine, and nifedipine) have been subjected to forced photodegradation in different containers [29]. For this purpose, bottles in transparent or amber glass and in transparent or colored polyethylene terephthalate (PET) with different thicknesses were tested. As a comparison, a quartz container, transparent to all visible and ultraviolet radiation, was used.

The stressing photodegradation tests were performed according to the ICH standard rules, using a xenon lamp as a radiant source. The qualitative variations of the drugs were monitored by UV spectrophotometry while the processing of the spectral data was performed via chemometric analysis multivariate curve resolution (MCR), able to resolve the spectral signals of all the components involved in the photodegradation process and estimate over time their quantitative determination [30].

The stability tests on the samples in amber glass showed different results for the various drugs. The protective effect for felodipine and nifedipine was minimal, while a marked increase in stability was recorded for lercanidipine and nimodipine. The degradation rate in PET was practically superimposable to that recorded in glass except for felodipine, showing a degradation rate 11 times lower than in the amber glass. Even better results were obtained with the adoption of containers in colored PET and in particular in blue PET, whose solution remained almost unchanged up to 6 hours of stressing irradiation. The authors attributed the photoprotective effect of the colored polymers to the overlaying of the absorption bands of the dyes and the wavelengths responsible for photodegradation.

Nifedipine solutions in polyethylene glycol 400, glycerine and mint oil have been monitored by HPLC over a period of 35 days under exposure to fluorescent light in amber glass bottles or in the same bottles wrapped in aluminum foil [31]. The amber glass samples protected by the aluminum foil retained more than 90% of the starting drug amount, while the use of amber glass lost more than 20% of the initial nifedipine concentration after 7 days.

The stability of isradipine in simple syrup has been studied when stored in amber glass bottles at 4 °C. The samples were analyzed by HPLC immediately after preparation and after 7, 16, 22, 28, and 35 days. Ninety-five percent of the initial isradipine concentration remained throughout the study period in all samples [32].

The protection of photosensitive drugs through the adoption of appropriate containers and packaging material must be considered from different points of view. The costs associated with packaging can be inconvenient for the production company. In some cases, packaging materials may provide insufficient protection. Furthermore, the need to inspect solutions, especially for sterile, clear and colorless liquid products, severely limits the use of dark or opaque bottles [33]. These are among the main reasons why the research is moving towards the definition of new approaches to photostabilize the drugs.

Table 1 lists the papers dealing with the adoption of various containers for DHP drug solutions.

### 2.2. Use of Light-Absorbing Excipients

The addition of excipients with large absorption regions is another system that has aroused interest from researchers in the field of photoprotection. The protective principle is based on the superposition of the absorption spectra of these excipients with those of the drugs. However, studies in this field are still being tested and have not yet produced satisfactory results.

In the case of DHP drugs, good results have been obtained with the addition of excipients with anti-oxidant capacity. The photostability of nisoldipine in methanol solutions has been tested after the addition of β-carotene. Tests were conducted under stressful conditions, using a high-pressure mercury lamp as an irradiation source, and the samples were monitored by UV spectrophotometry and HPLC. The degradation rate of nisoldipine decreased with increasing β-carotene concentration, confirming the role of this excipient as a photoprotective agent [34].

An interesting proposal for photoprotective formulations is the preparation of *liquisolid* solutions [35]. This approach has been tested for amlodipine by preparing different formulations with propylene glycol mixed with water in a 1:1 drug ratio, Avicel PH 102, amorphous silicon, and titanium dioxide. The preparations were subjected to various radiant energies and the residual drug reached 97.37% compared to 73.8% of the medicinal substance in a simple solution after 8 hours of light irradiation.

The use of photo-absorbing substances for the photoprotection of DHP drugs is quite limited, probably due to the difficulty related to interference with formulations. Table 2 shows the works on this topic.

### 2.3. Incorporation in Liposomes

**Figure 2:**
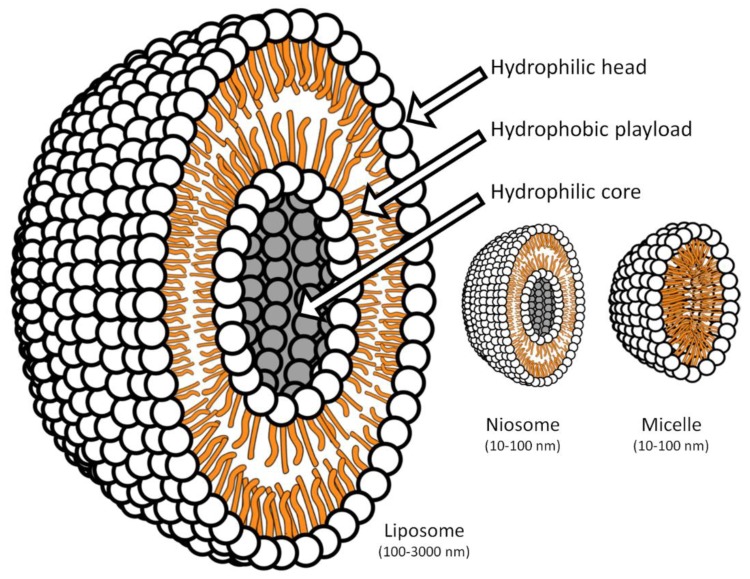
Scale representation of the central section of liposomes, niosomes and micelles.

Liposomes are complex structures formed by one or more double lipid layers that self-assemble to form hollow microspheres, able to incorporate different classes of substances, including drugs, through non-covalent bonds [36,37,38,39]. Liposomes usually are between 100 and 3000 nanometers in diameter. Niosomes (non-ionic liposomes) differ from “classic” liposomes because the phospholipidic layers are replaced by non-ionic synthetic amphiphilic lipids, usually added to cholesterol. The niosomes are smaller than 200 nanometers, are very stable and have various peculiar characteristics that, inter alia, make them very suitable for topical use. Figure 2 shows the split of the most used micellar systems.

Several studies have been published on the incorporation of DHP drugs into liposomes, in order to increase their solubility and photostability. The photoprotective ability of the complexes resulting from the incorporation of a series of DHP drugs into liposomes has been proposed in 2006 by Ragno et al [40]. Satisfactory results were obtained for most of the drugs tested, except for manidipine and lercanidipine. The worst results for these two compounds were associated with the difficulty of including these molecules in the micellar system.

In another work, the photostability of amlodipine was tested after incorporation into liposomes, exposing the liposomal complexes to stressed conditions of irradiation equal to 11,340 kJ m^−2^, in accordance with the ICH rules. The formulations were able to significantly increase the stability of the drug [41]. Also, to improve the photostability of amlodipine, a dry oil-in-water emulsion was prepared using labrafil M1944CS and dextrin. Overall, 94.4% of the drug remained intact after 24 hours of UV irradiation against 33.1% in powder [42]. Naturally, the micellar formulation, compared to the solid composition, also greatly improved the bioavailability of amlodipine.

Incorporation into liposomes for felodipine and nimodipine has still been proposed using ionic or non-ionic surfactants, such as sodium dodecyl sulfate, dodecyl pyridinium chloride, and mono lauryl sucrose ester. This work aimed primarily at studying the influence of surface charge in modulating the photo-reactivity of these drugs. This study showed that the drugs were positioned near the interface of the liposomal matrix without affecting the photodegradation rate of the molecules [43,44].

Niosomal formulations loaded with lacidipine through a thin film hydration technique were optimized by studying four factors: vesicle size, entrapment efficiency, flux and skin permeation. These niosomal vesicles have demonstrated to be an efficient nano-vesicular vehicle for the transdermal release of lacidipine, with excellent permeation values. The stability studies have shown great stability, not observing significant variations [45].

The works that involve DHP drug incorporation into self-assembling micellar systems are summarized in Table 3.

### 2.4. Incorporation in Cyclodextrins

Cyclodextrins are a family of cyclic oligosaccharides, consisting of multiple glucopyranose units joined through alpha 1-4 glycosidic bonds. Depending on the number of glycopyranosilic units, cyclodextrins are distinguished in α-cyclodextrins consisting of six carbon units; β-cyclodextrins consisting of seven carbon units; and γ−cyclodextrins consisting of eight carbon units. In recent years, cyclodextrins have been used in various fields. One of the most interesting applications is the transport of active ingredients within the human organism. In fact, the peculiar ring structure allows to create a hydrophobic core, useful for conveying lipophilic ingredients, and an extremely hydrophilic external environment, fundamental for improving solubility, absorption and bioavailability of active ingredients [46,47,48,49].

Since 1993, our research group has studied the stability of DHP drugs under light and has then begun to define photostabilization approaches [50]. Nifedipine, which is the lead of this class of drugs, and has a very high photosensitivity, was significantly more stable after complexation with hydroxypropyl-cyclodextrin and dimethyl-cyclodextrin. In an interesting work of 1997, after demonstrating the effectiveness of complexation with cyclodextrin in increasing light stability, the influence of the substituents on the phenyl ring was analyzed [51]. Photodegradation was shown to increase significantly in the presence of a nitro group. 

A series of 11 DHP drugs were incorporated into cyclodextrin and the photoprotective capacity of the formed complexes was monitored [40]. Significant photostability was demonstrated for amlodipine, felodipine, nisoldipine and nitrendipine. The other compounds showed 10% degradation in less than 10 minutes and a half-life of less than 1 hour. The different results were attributed to the efficiency of drug entrapment in the cyclodextrins, probably caused by the molecular volume. In fact, lercanidipine and manidipine, which have large chemical groups on the two molecular rings, did not show significant variation in the degradation rate.

The photostability of amlodipine was monitored in inclusion systems based on cyclodextrin. The residual concentration detected after a radiant exposure of 11,340 kJ m^−2^ was 90%, better than the value shown by commercial tablets [41]. Solubility and photostability of felodipine was studied when incorporated into (2-hydroxypropyl)-β-ciclodextrin. The influence of both parameters was evaluated in the presence of water-soluble polymers such as polyvinylpyrrolidone, polyethylene glycol, and hydroxypropylmethylcellulose. The solubility of the drug in cyclodextrin increased significantly and the degradation rate of the complex was significantly reduced [52].

The stability of barnidipine was also studied after incorporation into cyclodextrin. After a forced irradiation exposure of 225 kJ m^−2^, the residual concentration of the drug was still 72%. The photostability of the drug was almost complete when the cyclodextrin-drug complex was in turn trapped in the liposomes [53].

Isradipine photostability studies by incorporation into cyclodextrins has been described in various papers. The formation of the drug-cyclodextrin complexes also aimed to promote the water solubility of the drug, since this is very slightly soluble. The β-cyclodextrin drug complex was incorporated into prolonged-release hydroxypropyl methylcellulose tablets and monitored by differential scanning calorimetry and FTIR. The formulation was stable for up to four days of exposure [54]. In another work, the isradipine–β-cyclodextrin complex was tested for the development of formulations capable of being absorbed by the buccal membrane. Isradipine in these complexes was more stable than pure drug [55]. Isradipine was also incorporated into methyl-β-cyclodextrin and its photostability was doubled compared to pure drug [56].

The photosensitivity of nicardipine complexed with cyclodextrin in aqueous solution was tested during exposure to UV A and B radiation. Photostability was significantly increased when the drug was incorporated into β-cyclodextrin, γ-cyclodextrin, hydroxypropyl-α-cyclodextrin, hydroxypropyl-β-cyclodextrin, hydroxypropyl-γ-cyclodextrin, (2-hydroxyethyl)-β-cyclodextrin, and methyl-β-cyclodextrin, while the complex with α-cyclodextrin was on the contrary more photolabile. This study was carried out on both the enantiomers of nicardipine, detecting a clear difference in the profiles of photodegradation and related kinetic constants [57].

The complexation of manidipine with cyclodextrin reduces the rate of photodegradation of the nitro-phenylpyridine derivative and the corresponding nitrate [58]. 

Table 4 lists the various approaches proposed to minimize the photodegradation of DHP drugs by incorporation into cyclodextrins.

### 2.5. Cyclodextrins in Liposomes

The photostability of barnidipine complexed with cyclodextrin and in turn incorporated into a liposomal matrix has shown good results. The residual concentration of the drug in the cyclodextrin-in-liposomes matrices reached the value of 90.78% of residual drug after a radiation exposure of 225 kJ m^−2^, more than the values recorded when the drug was incorporated only in liposomes or complexed with cyclodextrin [53].

### 2.6. Microspheres and Nanocapsules

In recent years, great interest has been achieved by the incorporation systems of drugs represented by microspheres or polymer-based nanocapsules. The microspheres have been tested for controlled drug delivery, allowing a significant reduction in doses and a reduced frequency of administration. In the field of DHP drugs, study has been mainly focused on the lead nifedipine.

Nifedipine was incorporated into polymer nanocapsules formed by surfactants pluronic F68 and polyvinyl alcohol to preserve the drug from degradation. The efficiency of these formations in protecting the drug from light was tested by registering a significant protection [59]. The photostability of nifedipine in a solid dispersion formulation was also evaluated by dispersing the drug in porous calcium silicate. The degree of photoprotection was increased if compared to a classic microcrystalline cellulose formulation, probably also due to the greater physical shielding against the light that calcium silicate offers [60]. Nifedipine was also complexed with weak cation exchange resins, indion 204 and indion 264. The Indion 204 resin was found to be a very valid complexing agent for reducing the photosensitivity of nifedipine [61].

The adoption of micro and nanosystem for the photoprotection of DHP drugs are listed in Table 5.

## 3. Conclusions

The current international law provides that registration and introduction of a new drug on the market is preceded by a series of tests to ascertain its scientific and technical parameters. Among the various tests, an important role is the investigation of the behavior of the drug when exposed to light. This parameter has become necessary after the discovery of a high number of photosensitive drugs whose degradation can lead to the loss of therapeutic activity or to the formation of toxic products. Drugs belonging to the 1,4-dihydropyridine class, which are currently the most widely used in antihypertensive therapy, have a marked sensitivity to light, which in most cases leads to oxidation of the dihydropyridine ring to pyridine, with a complete loss of pharmacological action.

Because of the strong photosensitivity of these drugs, almost all the specialties are nowadays in solid form, well protected by blisters not transparent to light. However, many studies aim to define different means of protection for liquid formulations. This review collects the most modern studies on the chemical or physical means adopted, or still being tested, to minimize the effects of light on the DHP drugs. Some studies aim for the synthesis of new DHP drugs with a molecular structure more resistant to the common processes of photodegradation of these drugs. In this case, very interesting are the definition of QSPR mathematical models that correlate the molecular substituents of the pharmacophore to the photosensitivity of the molecules. Many studies seek instead alternatives to the use of dark or coated glass with the design of new photoprotective systems. Among these, the most studied are supramolecular matrices based on cyclodextrins or micellar systems, or use of excipients capable of absorbing wavelengths in visible or UV regions and incorporation into micro and nanomolecular systems.

## Figures and Tables

**Table 1:** Use of dark or opaque containers. MCR: multivariate curve resolution.

DHP Drugs	Photoprotection Method	Analytical Approach *	References
Felodipine	Transparent glass, amber glass, transparent PET, colored PET, quartz containers	UV, MCR	[29]
Lercanidipine			
Nifedipine			
Nimodipine			
Nifedipine	Polyethylene glycol 400, glycerine and mint oil solutions in amber glass bottles, amber glass bottles wrapped in aluminum foil	HPLC	[31]
Isradipine	Solutions in simple syrup, amber glass bottles at 4 °C	HPLC	[32]

* Ultraviolet Spectroscopy (UV); High Performance Liquid Chromatography (HPLC).

**Table 2:** Use of light-absorbing excipients.

DHP Drugs	Photoprotection Method	Analytical Approach *	References
Nisoldipine	Addition of β-carotene	UV, HPLC	[34]
Amlodipine	Formulations with propylene glycol mixed with water in a 1:1 drug ratio, Avicel PH 102, amorphous silicon, and titanium dioxide	X-ray, FT-IR	[35]

* Ultraviolet Spectroscopy (UV); High Performance Liquid Chromatography (HPLC); Fourier-Transform Infrared Spectroscopy (FT-IR).

**Table 3:** Use of liposome matrices.

DHP Drugs	Photoprotection Method	Analytical Approach *	References
Amlodipine Felodipine Isradipine Lacidipine Lercanidipine Manidipine Nicardipine Nifedipine Nimodipine Nisoldipine Nitrendipine	Incorporation in phosphatidyl choline liposomes	UV	[40]
Amlodipine	Incorporation in phosphatidyl choline liposomes	UV	[41]
Amlodipine	Dry oil-in-water emulsion prepared with labrafil M1944CS and dextrin	UV	[42]
Felodipine Nimodipine	Liposomes with ionic or non-ionic surfactants (sodium dodecyl sulfate, dodecyl pyridinium chloride, mono lauryl sucrose ester)	UV, HPLC	[43]
Lacidipine	Niosomal formulated through a thin film hydration technique	UV	[45]

* Ultraviolet Spectroscopy (UV); High Performance Liquid Chromatography (HPLC).

**Table 4:** Use of cyclodextrin matrices.

DHP Drugs	Photoprotection Method	Analytical Approach *	References
Nifedipine	Complexation with hydroxypropyl-cyclodextrin and dimethyl-cyclodextrin	UV	[51]
Amlodipine Felodipine Isradipine Lacidipine Lercanidipine Manidipine Nicardipine Nifedipine Nimodipine Nisoldipine Nitrendipine	Incorporation in methyl-β-cyclodextrin	UV	[40]
Amlodipine	Incorporation in methyl-β-cyclodextrin	UV	[41]
Felodipine	Incorporation in 2-hydroxypropy-β-cyclodextrin in the presence of water-soluble polymers	X-ray, DSC, FT-IR	[52]
Barnidipine	Complexation with α-cyclodextrin, β-cyclodextrin, methyl-β-cyclodextrin, hydroxypropyl-β-cyclodextrin, γ-cyclodextrin	UV	[53]
Isradipine	Incorporation of the β-cyclodextrin drug complex into prolonged-release hydroxypropyl methylcellulose tablets.	DSC, FT-IR	[54]
Isradipine	Complex with beta-cyclodextrin	X-ray, DSC	[55]
Isradipine	Complex with methyl-β-cyclodextrin	UV, HPLC	[56]
Nicardipine	Incorporation in β-cyclodextrin, γ-cyclodextrin, hydroxypropyl-α-cyclodextrin, hydroxypropyl-β-cyclodextrin, hydroxypropyl-γ-cyclodextrin, (2-hydroxyethyl)-β-cyclodextrin and methyl-β-cyclodextrin	Capillary electrophoresis	[57]
Manidipine	Complex with β-cyclodextrin	UV, HPLC-MS	[58]

* Ultraviolet Spectroscopy (UV); High Performance Liquid Chromatography – Mass Spectroscopy (HPLC-MS); Differential Scanning Calorimetry (DSC); Fourier-Transform Infrared Spectroscopy (FT-IR).

**Table 5:** Incorporation in microsystems.

DHP Drugs	Photoprotection Method	Analytical Approach	References
Nifedipine	Polymer nanocapsules by multichonic surfactants F68 and polyvinyl alcohol	HPLC	[59]
Nifedipine	Solid formulation by dispersing the drug in porous calcium silicate	HPLC	[60]
Nifedipine	Complex with weak cation exchange resins, indion 204 and indion 264	UV	[61]
